# The Impact of the Extent of Surgery on the Long-Term Outcomes of Patients with Low-Risk Differentiated Non-Medullary Thyroid Cancer: A Systematic Meta-Analysis

**DOI:** 10.3390/jcm9072316

**Published:** 2020-07-21

**Authors:** Andreea Bojoga, Anna Koot, Johannes Bonenkamp, Johannes de Wilt, Joanna IntHout, Peep Stalmeier, Rosella Hermens, Johannes Smit, Petronella Ottevanger, Romana Netea-Maier

**Affiliations:** 1 National Institute of Endocrinology “C.I. Parhon”, 011863 Bucharest, Romania; coriiuandreea@gmail.com; 2 Radboud Institute for Health Sciences, Department for Health Evidence, Radboud University Medical Center, 6500 HB Nijmegen, The Netherlands; joanna.inthout@radboudumc.nl (J.I.); peep.stalmeier@radboudumc.nl (P.S.); 3 Department of Internal Medicine, Division of Endocrinology, Radboud University Medical Center, 6525 GA Nijmegen, The Netherlands; jan.smit@radboudumc.nl (J.S.); romana.netea-maier@radboudumc.nl (R.N.-M.); 4 Department of Surgical Oncology, Radboud University Medical Center, 6525 GA Nijmegen, The Netherlands; han.bonenkamp@radboudumc.nl (J.B.); hans.dewilt@radboudumc.nl (J.d.W.); 5 Scientific Institute for Quality of Healthcare (IQ Healthcare), Radboud University Medical Center, 6500 HB Nijmegen, The Netherlands; rosella.hermens@radboudumc.nl; 6 Department of Internal Medicine, Division of Oncology, Radboud University Medical Center, 6525 GA Nijmegen, The Netherlands; nelleke.ottevanger@radboudumc.nl

**Keywords:** low-risk thyroid cancer, thyroid lobectomy, total thyroidectomy, shared decision making

## Abstract

Recently, the management of patients with low-risk differentiated non-medullary thyroid cancer (DTC), including papillary and follicular thyroid carcinoma subtypes, has been critically appraised, questioning whether these patients might be overtreated without a clear clinical benefit. The American Thyroid Association (ATA) guideline suggests that thyroid lobectomy (TL) could be a safe alternative for total thyroidectomy (TT) in patients with DTC up to 4 cm limited to the thyroid, without metastases. We conducted a meta-analysis to assess the clinical outcomes in patients with low-risk DTC based on the extent of surgery. The risk ratio (RR) of recurrence rate, overall survival (OS), disease-free survival (DFS) and disease specific survival (DSS) were estimated. In total 16 studies with 175,430 patients met the inclusion criteria. Overall, low recurrence rates were observed for both TL and TT groups (7 vs. 7%, RR 1.10, 95% CI 0.61–1.96, *I*^2^ = 72%), and no statistically significant differences for OS (TL 94.1 vs. TT 94.4%, RR 0.99, CI 0.99–1.00, *I*^2^ = 53%), DFS (TL 87 vs. TT 91%, RR 0.96, CI 0.89–1.03, *I*^2^ = 85%), and DSS (TL 97.2 vs. TT 95.4%, RR 1.01, CI 1.00–1.01, *I*^2^ = 74%). The high degree of heterogeneity of the studies is a notable limitation. Conservative management and appropriate follow-up instead of bilateral surgery would be justifiable in selected patients. These findings highlight the importance of shared-decision making in the management of patients with small, low-risk DTC.

## 1. Introduction

Differentiated non-medullary thyroid cancer (DTC) is the most common endocrine malignancy and accounts for 85% of thyroid cancer [1]. Epidemiological studies have shown that its incidence has increased continuously worldwide over the past three decades, which has been largely attributed to the diagnosis of small, often incidentally detected tumors, due to improvement and wider use of diagnostic techniques [2,3,4,5]. DTC includes papillary thyroid carcinoma (PTC) and follicular thyroid carcinoma (FTC) subtypes, with PTC representing more than two thirds of the DTC [6,7]. The majority of patients with DTC have a favorable prognosis with an excellent long-term overall survival rate, with less than 10% of patients being expected to die of the disease in 10 years or more [7,8,9,10,11].

There is controversy on the optimal treatment of patients with DTC, raising the question whether some of the patients with low-risk DTC might be overtreated and therefore at risk for complications of these treatments without having a clear benefit in terms of oncological outcome. Until recently, total thyroidectomy (TT) followed by postoperative radioactive iodine (RAI) treatment to ablate the thyroid remnant tissue or as an adjuvant therapy was recommended for the majority of patients by many international guidelines. Over the past decade, clinical practice has shifted towards a more individualized approach. In particular, better understanding of the biology and excellent outcomes of these tumors has led to recommending a more conservative approach for low-risk patients [12]. TT is no longer recommended for patients with radically removed microcarcinoma as it is recognized that these tumors have an excellent outcome and prospective and retrospective evidence suggests that many of the patients with indolent papillary microcarcinomas might even be followed-up conservatively [13,14]. However, for the patients with low-risk DTC, i.e., with tumors between 1 and 4 cm, there is more controversy regarding the extent of surgery. While the majority of publications have not found the extent of surgery to impact on the survival of these patients, some report a higher risk for recurrent disease in thyroid lobectomy (TL) [15,16,17,18].

The role of conservative limited surgery versus more extensive surgery for patients with low-risk DTC has not been studied in prospective randomized controlled trials, as large sample size and a long-term follow-up would be required [19,20]. Therefore, recommendations and practice are based on retrospective data, which bare inherent sources of collection and analytical bias [21,22]. Moreover, the low-risk category has been differently defined in various studies. The most recent guideline of the American Thyroid Association (ATA) recommends that TL could be a safe alternative for TT in the treatment of patients with unifocal DTC up to 4 cm in diameter limited to the thyroid, without preoperatively known risk factors such as suspicious lymph nodes on the preoperative ultrasound and without distant metastases at presentation [23].

The purpose of this meta-analysis is to compare the clinical outcomes in adult patients with low-risk DTC based on two surgical modes, TL versus TT, and discuss in that light the arguments for and against TL and TT in low-risk DTC according to the definition and recommendation of the ATA.

## 2. Materials and Methods

The meta-analysis was performed in accordance with the Preferred Reporting Items for Systematic Reviews and Meta-analysis (PRISMA) guidelines [24]. We searched for relevant studies up to May 2019 using MEDLINE (Pubmed) database. Additional studies were identified by hand searching references in original articles and review articles. All studies selected for the meta-analysis met the following criteria: (1) articles reporting comparison outcomes for TL and TT in patients with “low-risk” DTC (PTC and FTC subtypes) regardless of the definition used and (2) the outcomes of interest were recurrence rates, overall survival (OS), disease free survival (DFS) and/or disease specific survival (DSS). In the analysis, recurrences were considered as indicated in the individual studies and defined exclusively as new structural recurrences (either in the thyroid bed or in the nonremoved thyroid tissue, the (cervical) lymph nodes, or distant metastases) confirmed by imaging and/or pathological examination. The major exclusion criteria were: (1) abstracts, reviews, age ≤ 18, non-English language, (2) duplicate data, (3) no reported outcomes mentioned, (4) studies focusing exclusively on microcarcinoma, (5) with less than 50 patients per arm. Patients were classified using various scoring systems (AJCC/TNM, AMES, ATA). A comparison between different scoring systems and editions of AJCC/TNM applied in included studies to define “low-risk” can be seen in Tables S1 and S2 Comparison of the AJCC UICC 3rd, 6th, 7th and 8th edition [23,25,26,27,28]. Low-risk AMES score is equivalent to stages I and II according to the TNM/AJCC staging system, [29] and patients included in the low-risk group will be TNM/AJCC stage I or II depending on their age, tumor size and lymph node status, having a proven high survival rate [12,26]. For the meta-analysis, we selected patients described as having tumors between 1–4 cm limited to the thyroid, without evidence of locoregional or distant metastases. The search terms were as follows: MeSH terms: “thyroid neoplasms”, “thyroidectomy” and keywords: “non-medullary thyroid neoplasm”, “thyroid surgery”, “lobectomy”, “low risk thyroid carcinoma” (the detailed presentation of the search procedure is available in Table S3).

### 2.1. Data Extraction

Two authors (A.B. and A.K.) extracted all relevant data independently. The following data were extracted: year of publication, first author’s name, country of study origin, % female, mean age, study design, surgical approach, follow-up duration, use of radioactive iodine (RAI), inclusion of high-risk patients and outcomes assessed. The data were reviewed by a third author (R.N.-M.). Disagreements were resolved through consensus.

### 2.2. Risk of Bias Assessment

We used a component approach to assess risk of bias for all included studies. The components were subdivided into five separate domains based on the Cochrane Risk-of-Bias In Non-Randomized Studies-of Interventions (ROBINS-I tool) [30]. The following domains and components, which could potentially bias a reported association between extent of surgery and outcome in thyroid cancer patients, were included [31].

### 2.3. Pre-Intervention

Domain 1: Bias due to confounding (see below)
Domain 2: Bias in selection of participants into the study
Inclusion of patients (consecutive inclusion of all patients eligible or a random sample is considered low risk of bias).
Definition of low risk thyroid cancer?

### 2.4. At Intervention

Domain 3: Bias in classification of intervention.
Were intervention groups clearly defined?

### 2.5. Post Intervention

Domain 4: Bias due to missing data
TNM-stage available (see below)
Reporting of high-risk patients (see below)
Reporting of RAI treatment (see below)
Reporting number of patients included per treatment arm (see below)
Domain 5: Bias in measurement of outcomes
Loss to follow-up (<5% is considered low risk of bias)
Criteria for extent of surgery (see below)
Reporting of outcome definition

Matched cohorts reported no bias of confounding. As the type of surgery procedure was not always clearly described, adequately reporting the criteria used for the extent of surgery was considered a low risk of bias. Availability of TNM-stage was considered adequate if the article mentioned T1–T4. Reporting of high-risk patients was considered adequate if the article mentioned T3–T4, N1 and/or M1 stage or mentioned numbers of high-risk patients with well-defined criteria for high risk. Reporting of RAI treatment was considered adequate if the numbers of patients receiving RAI treatment were mentioned. Reporting the number of patients included per treatment arm was considered adequate if the article mentioned the number of patients for each type of surgery.

### 2.6. Statistical Analysis

Pooled RR’s, 95% confidence intervals (95% CI) and 95% prediction intervals were calculated using random effects models (the inverse variance method, empirical Bayes estimator for τ^2^ and continuity correction of 0.5 in studies with zero cell frequencies). Heterogeneity among studies was evaluated using the *I*^2^ statistic [32]. We present the median proportion in the studies as the proportion per group. Subgroup analyses were conducted according to design, sample size, year of publication and duration of follow-up after TL and TT, to assess their potential contributions to outcomes. R version 3.5.3, with package meta version 4.9-5 was used for the analysis of the data [33,34].

Sensitivity analyses were performed to estimate the influence of each individual study on the overall risk ratio (RR). Studies were considered to have a low risk of bias if they met at least ten (out of eleven) of the above-mentioned criteria for low risk of bias. Articles were considered intermediate risk of bias if they met up to nine of the above-mentioned criteria for risk of bias. The potential publication bias was evaluated with a funnel plot [35].

## 3. Results

### 3.1. Characteristics of the Selected Studies

**Figure 1:**
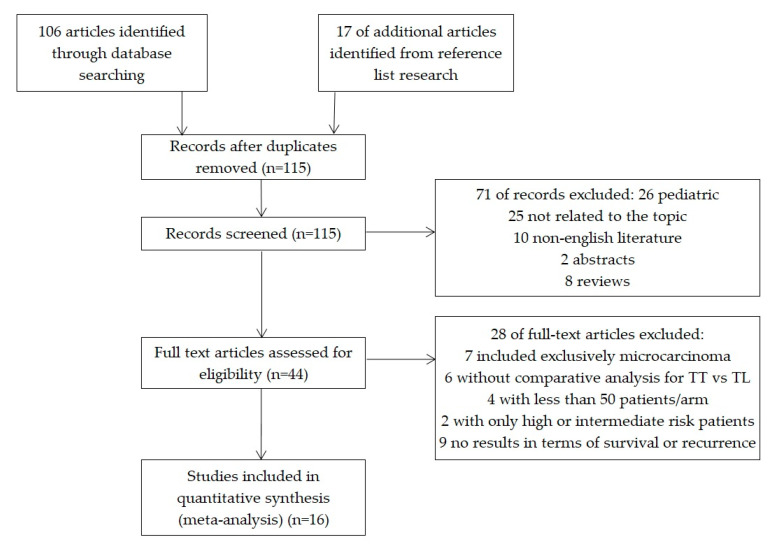
Flow chart for study selection.

In total, 106 abstracts and titles were obtained through electronic search, and 17 articles identified through manual search. The detailed search yielded no randomized controlled trials. Forty-four articles were found eligible, of which the results of 23 articles were examined in detail; 16 studies met the inclusion/exclusion criteria, which resulted in 175,430 patients. Figure 1 shows the details of the study selection process and the exclusion criteria. Characteristics of the 16 included studies are listed in Table 1.

### 3.2. Any Thyroid Cancer-Related Recurrence

**Figure 2:**
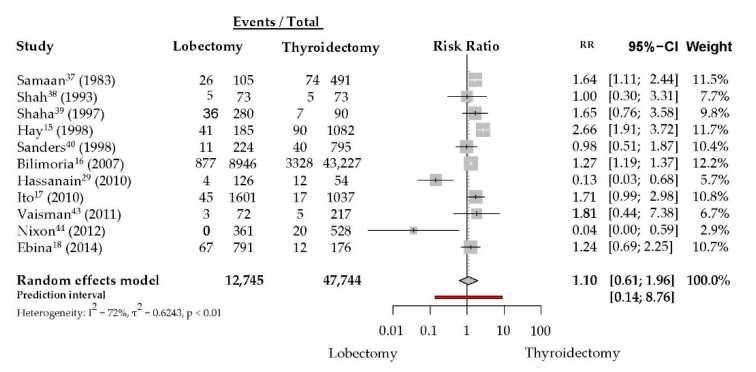
Recurrence rates of differentiated thyroid carcinoma after total thyroidectomy versus lobectomy.

The meta-analysis of any DTC-related recurrence was based on 60,534 patients in 11 studies with a median follow-up of 10 years. Median recurrence rates were 7% in the TL group and 7% in the TT group. Most of the studies only described overall recurrence rates, compared by extent of surgery [16,17,29,36,42]. Two studies also included recurrence rates for distant metastases [37,39]. Four of the included studies reported the local, regional and distant metastases recurrence rates [15,18,38,43]. Overall, similar tumor recurrence rates were observed for the TL group and the TT group (RR = 1.10, CI 0.61 to 1.96, *I*^2^ = 72%) (Figure 2). No significant difference was observed between the two surgical methods. There was a marked statistical heterogeneity. The subgroup analyses showed no significant impact of design, sample size, year of publication or time of duration on the results (data not shown).

### 3.3. Survival Outcomes

**Figure 3:**
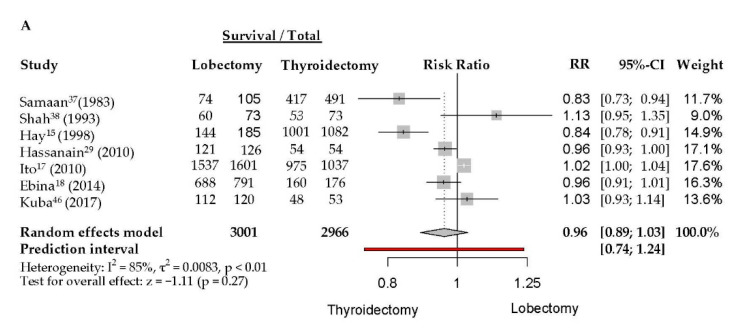

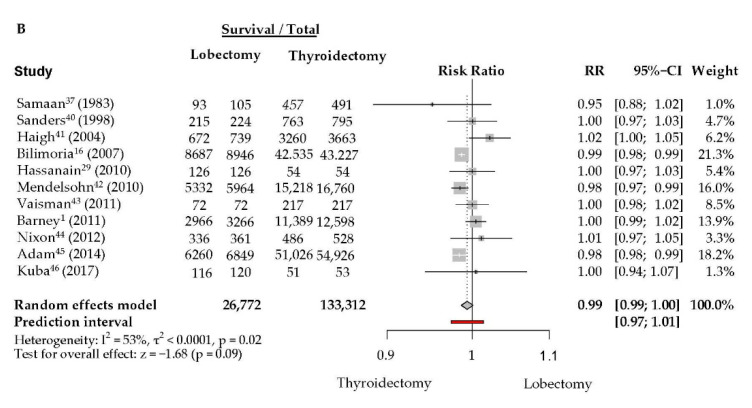

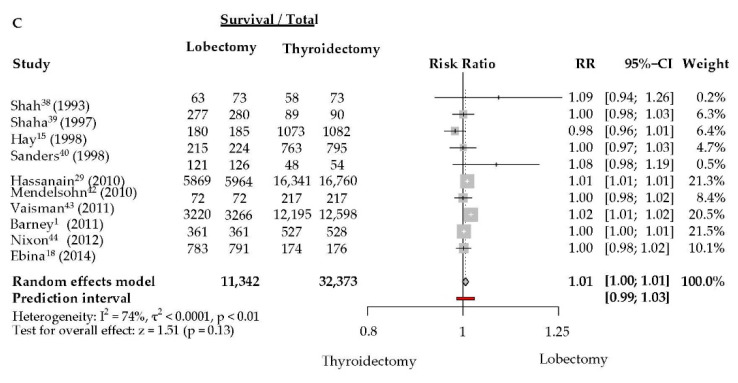
Survival rates of thyroid carcinoma after total thyroidectomy versus lobectomy. (**A**) DFS; (**B**) OS; (**C**) DSS.

The meta-analysis of DFS included 5967 patients in seven studies with a median follow-up of 10 years. Median DFS rates were 87% in the TL group and 91% in the TT group. No significant difference was observed between the two surgical methods for DFS (RR 0.96, CI 0.89 to 1.03, *I*^2^ = 85%). There was a marked statistical heterogeneity (Figure 3A).

The meta-analysis of OS was based on 160,084 patients in 11 studies with a median follow-up of 7 years. Median OS rates were 93% in the TL group and 93% in the TT group. There was a marked statistical heterogeneity. No significant difference was observed between the two surgical methods for OS (RR 0.99, CI 0.99 to 1.00, *I*^2^ = 53%). There was a marked statistical heterogeneity (Figure 3B).

The meta-analysis of DSS included 43,715 patients in 10 studies with a median follow-up of 11 years. Median DSS rates were 98% in the TL group and 99% in the TT group. No significant difference was observed between the two surgical methods for DSS (RR 1.01, CI 1.00 to 1.01, *I*^2^ = 74%). There was a marked statistical heterogeneity (Figure 3C).

### 3.4. Sensitivity Analysis

**Figure 4:**
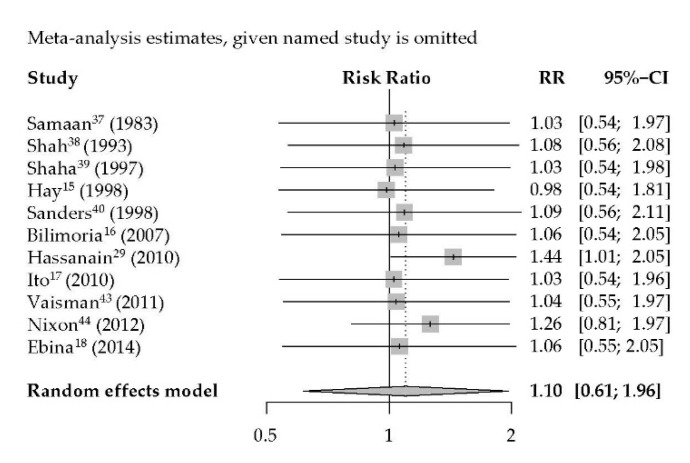
Sensitivity analysis for recurrence rates of well-differentiated thyroid carcinoma after thyroid lobectomy versus total thyroidectomy. DFS, OS and DSS showed no extreme effect and were not shown.

The results demonstrate that there is one individual study with an extreme effect on the overall RR for recurrence rates [29] (Figure 4).

### 3.5. Risk of Bias Assessment

Only two studies (13%) had a matched cohort and no bias of confounding. Definition of low-risk patients was adequately reported in 13 articles (81%). Inclusion of consecutive patients or use of a random sample was explicitly stated in 15 articles (94%). Loss to follow up was reported in six articles (37.5%), one of these six articles reported a loss to follow up of <5%. Four of these six articles also included patients who underwent less than a lobectomy or a subtotal thyroidectomy, procedures that are regarded as inferior in terms of oncologic outcome [29,38,39,46]. Some of the studies that clearly defined which of the patients had these insufficient surgeries also included a separate analysis for these [1,31,36,37,44]. Criteria for extent of surgery were met in 14 articles (87.5%). Reporting of outcome definition was adequate in 16 articles (100%). Availability of T-stage was adequate in 15 articles (94%). Reporting high risk patients was adequate in 12 articles (75%) (see also Table S4). Reporting of RAI treatment was adequate in 13 articles (81%) (see also Table S4). Reporting number of patients per treatment arm was adequate in 16 articles (100%).

One article showed low risk of bias (met ten out of eleven criteria), and five articles showed intermediate risk of bias (met nine out of eleven criteria). There was no study that met all eleven criteria. The risk of bias for each study is reported in Table S5.

### 3.6. Publication Bias

**Figure 5:**
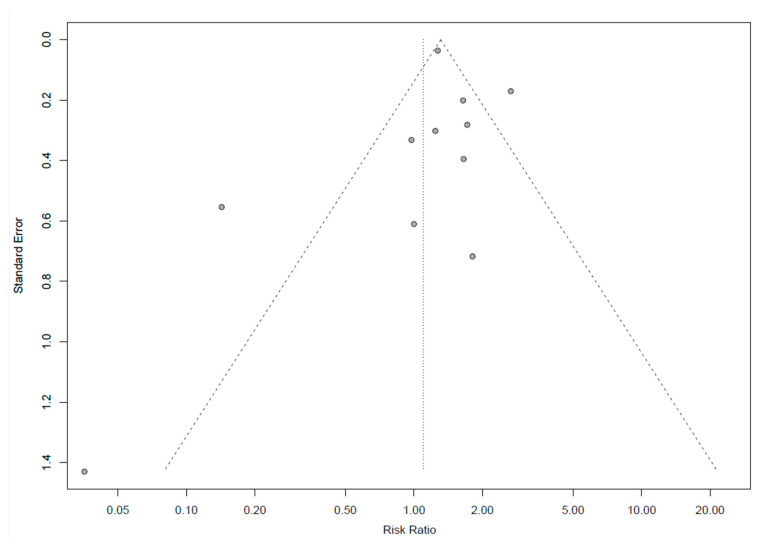
Funnel plot of recurrence rate for publication bias. Each point represents a separate study for the indicated association. OS, DFS and DFS had similar outcomes and are not shown.

Publication bias was examined by funnel plots. No clear indication of publication bias was observed for recurrence rates (Figure 5).

## 4. Discussion

To our knowledge, this study is the first meta-analysis focusing on the outcome of surgical extension in terms of recurrence and survival in patients with low-risk DTC 1–4 cm without clinical evidence of locoregional of distant metastases. Our meta-analysis shows no statistically significant differences between TL versus TT regarding DTC-related recurrence, DSS, OS and DFS rates.

The effect of the extent of surgery for patients with low risk DTC on patients’ outcome has long been a matter of discussion partly because of a lack of evidence-based randomized data from prospective studies. The early study of Mazzaferri et al. showed a statistically significant advantage of TT in comparison to TL regarding both recurrence and survival [47]. These results were later supported by Bilimoria et al., who showed, in a large retrospective analysis of 52,173 patients in the National Cancer Database (NCDB), that lobectomy resulted in higher risk of recurrence and death for tumors larger than 1 cm [16]. Nonetheless, the study of Mazzaferri was done before the era of contemporary risk stratification and since then preoperative evaluation and follow-up has improved [47]. Moreover, Bilimoria et al. presented only data on OS and lacked significant data about potential high-risk features [16]. Therefore, a critical reappraisal was done by Adam et al., who applied an analysis of a more contemporary (1998–2006) NCDB cohort of patients. This study showed that OS was similar in patients undergoing TL versus TT for tumors 1–4 cm after multivariable adjustment for clinical and pathological factors [44]. Even in patients with high risk features like extracapsular invasion and clinical evidence of lymph node metastasis, Haigh et al. reported that extent of thyroidectomy had no notable effect on estimated survival [40]. Mendelsohn et al. showed that after controlling for tumor size, multivariate analysis revealed no survival difference between TT versus TL [41]. Other studies, not meeting the selection criteria for this meta-analysis, also report excellent long-term clinical outcomes in selected low-risk patients [46,48,49,50,51,52]. Our meta-analysis supports the lack of a significant effect of the extent of surgery on the survival outcome in the low-risk patient category amenable for TL according to the ATA guidelines.

Lower complication rate, avoiding iatrogenic hypothyroidism and shorter duration of surgery are important arguments for preferring TL. Nonetheless, there could be several reasons for preferring a TT. One reason is the concern regarding an increased risk for recurrent or residual disease (e.g., persistence of microscopic lymph node metastases or multifocal tumor localizations in the opposite thyroid lobe). In addition, distant metastases could be missed and might only become apparent on whole body scans performed after ablative treatment with RAI, as limited surgery is usually not followed by treatment with RAI. In this meta-analysis, both local and distant metastases recurrence rates were low. There was no statistically significant difference of local recurrences in TL compared to TT, and distant metastases recurrence rates were higher in TT. Of note, only five studies [17,18,29,36,45] mentioned ultrasound as a routine preoperative assessment but only in patients evaluated since 2005 [43], while all studies also included patients who were treated before 2005. Thus, malignancies in the contralateral remnant lobe and small cervical lymph node metastases might have been missed, resulting in suboptimal treatment in these patients.

A second reason for preferring TT is that bilateral tumor localizations are found in 20% to 85% of patients with PTC after total or completion thyroidectomy, but the majority are considered occult carcinoma [7,53,54]. Proponents of TT argue that this feature is associated with a higher risk of recurrence and thus, reoperation [36,54]. Mazzaferri and Huang have shown that in patients with three or more foci of PTC or FTC (any size), there was a higher cancer-related mortality rate, but this association was not present after adjustment for multivariate analysis [21]. Other studies do not report an association between tumor multicentricity and prognosis, thus the clinical significance of occult carcinoma is questionable [39,53,55,56]. Of the studies included in this meta-analysis that mentioned multicentricity, none showed an association between multicentricity and prognosis [17,18,29,37,44,45].

A third reason is that microscopical lymph node metastases are also likely to be found in histopathological samples (up to 90% in the central compartment and up to 40% in the lateral compartment) [7], but these remain quiescent in the vast majority of patients, with only 10% developing clinically significant disease [7,12]. The risk of persistent micro-nodal disease also persists in patients who had TT if prophylactic lymph node dissection has not been performed, a procedure that is not routinely done in patients without clinical or radiological evidence of lymph node metastases. Because of the lack of prospective studies, a conservative surgical approach should be complemented with radiological follow-up by neck ultrasound, preferably performed by expert radiologists in order to detect the small volume nodal disease that might develop into gross recurrent disease requiring additional treatment. Our analysis indicates that even if the risk of recurrent disease in low-risk patients is present, this risk remains low regardless of the extent of surgery, and with proper management it does not impact the long-term survival of the patients. Moreover, it has been argued that these recurrent tumors are amenable for salvage surgery and therefore a “stepwise” approach to therapy of these patients could be an acceptable alternative for the minority of patients in which the disease recurs, while the limited surgery might suffice for the oncologic control of the remaining low-risk patients [23].

A fourth and important reason to prefer TT over TL is routine use of RAI ablation in the majority of patients with tumors 1 cm or greater. In terms of outcome, RAI ablation has not been shown to clearly benefit the low-risk group patients in the absence of any high-risk features, while being associated with some side effects [23,57,58]. However, RAI ablation facilitates follow-up by enabling the use of thyroglobulin (Tg) as a reliable marker of a favorable prognosis. Though the Tg levels are less reliable in patients who have undergone TL [59,60], Tg level trends combined with neck ultrasound can be effective follow-up methods for these patients as well [12,57,61].

Our study has some limitations which should be considered by the interpretation of the data. Due to the lack of randomized prospective studies, our estimates were based on non-randomized retrospective studies which are prone to selection bias, are limited by the availability of patient data and are characterized by a high degree of heterogeneity. The sensitivity analysis demonstrates that there is one individual study with an extreme effect on the overall RR for recurrence rates. Hassanain et al. showed a much higher recurrence rate in the more extensive surgery patients, which indicates selection bias [29]. Only two articles had no bias of confounding on the effect of intervention because of a matched cohort. Therefore only one article included had a low risk of bias and five articles had an intermediate risk of bias according to the Cochrane Risk-of-Bias In Non-Randomized Studies-of Interventions (ROBINS-I) tool [30]. Inclusion of high-risk patients, using various staging systems, inclusion of tumors larger than 4 cm (six studies, incomplete data in another three studies), use of other surgical interventions such as nodulectomy, local excision, and subtotal thyroidectomy, which are considered to be inappropriate for thyroid cancer treatment [62,63] (in 12 of the included studies) likely biased the outcome in favor of one surgical approach over the other. Furthermore, the comparison of these studies is influenced by adjuvant therapies such as RAI or external beam radiation therapy, routine thyroid stimulating hormone suppression, and lymph node dissection. RAI in particular was administered more often in the patients that had undergone TT, most likely reflecting the selection bias towards more unfavorable clinical features or the local protocols. However, this might rather have conferred a clinical benefit to those who had TT instead of TL. Notably, the two cohort studies [38,46] in which propensity score matching for clinical features of the cohorts of patients having TT and TL was done resulted in a similar outcome. Verburg et al. showed, in a recent systematic review on the potential benefits of RAI treatment in low-risk patients, that the results of different studies are mixed, largely because of multiple potential sources of bias and heterogeneity with respect to RAI treatment protocols and activities applied. This precludes, for the moment, a definitive answer to this question and strengthens the need for well-designed randomized clinical trials [64]. As time to disease recurrence in low risk DTC can be very long, mean follow-up period in some studies might not have been sufficient, thus affecting the survival and recurrence outcome as an end point. Nonetheless, the majority of studies included 10 to 20 years of follow-up, which is largely reassuring.

When selecting the optimal surgical extent in patients with low-risk DTC, the goal is to minimize the risk of death from disease and recurrence, while avoiding potential iatrogenic injury from overtreatment. Disease-related characteristics are critical, and risk stratification is paramount. Individual patient values and preferences, as well as surgeons’ experience and quality of follow-up and patients’ adherence to follow-up should also be considered when making this decision [65].

## 5. Conclusions

TL is a safe procedure for treatment of low-risk DTC patients, with excellent oncological outcomes and potentially lower treatment side effects. Initial risk assessment and ongoing dynamic risk stratification can reduce the uncertainty about disease persistence and individualize follow-up and treatment in order to reduce recurrence rate, the risk of completion thyroidectomy, and patient anxiety. However, it is important to inform the patients about the expected outcomes for both TL and TT with respect to morbidity, need for hormone replacement and the risk of reoperation. Thus, shared decision-making should be considered when the choice for the most appropriate surgical approach is being made.

## Figures and Tables

**Table 1:** Characteristics of the selected studies included in the meta-analysis.

Authors/Year of Publication	Country	Female (%)	Mean Age (Y)	Time of Surgery Stated	Study Design	Surgical Approach		Follow-Up	Outcomes Assessed	RAI Given (%)	Histological Subtypes Included	High Risk Patients Included (%)
						TL	TT					
Samaan 1983 [36]	USA	73	NA	1951–1975	Retrospective cohort study	105	491	6–30 Y	Recurrence, OS	yes (34%)	PTC, FTC, HCC	yes
Shah 1993 [37]	USA	62	57	1930–1980	Retrospective matched cohort study	73	73	20 Y	Recurrence, DFS	yes *	PTC, FTC	yes
Shaha 1997 [38]	USA	74	31	1930–1985	Retrospective cohort study	280	90	20 Y	Recurrence, DSS	NA	PTC, FTC	no
Hay 1998 [15]	USA	NA	43	1940–1991	Retrospective cohort study	185	1082	16 Y	Recurrence, DFS, DSS	yes	PTC	no
Sanders 1998 [39]	USA	72	44	1940–1990	Retrospective cohort study	224	795	0–47 Y	Recurrence, OS, DSS	NA	PTC, FTC, HCC	yes
Haigh 2004 [40]	USA	77	NA	1988–1995	Retrospective population-based cohort	739	3663	1 mo–12 Y	OS	yes	PTC	Yes **
Bilimoria 2007 [16]	USA	76	43	1985–1998	Retrospective population-based cohort	8946	43,227	5.8 Y	Recurrence, OS	yes	PTC	yes
Mendelsohn 2010 [41]	USA	78	44	1988–2001	Retrospective population-based cohort	5964	16,760	9.1 Y	OS, DSS	yes	PTC	yes
Hassanain 2010 [29]	Canada	73	NA	1982–2002	Retrospective cohort study	126	54	4–25 Y	Recurrence, DFS	yes (28.3%)	PTC, FTC	yes
Ito 2010 [17]	Japan	94	51.1 ± 12.4	1987–2005	Retrospective cohort study	1601	1037	0.5–34 Y	Recurrence, DSS	yes (0.1%)	PTC	no
Barney 2010 [1]	USA	NA	NA	1983–2002	Retrospective population-based cohort	3266	12,598	2 mo–19.9 Y	OS, DSS	yes	PTC, FTC	yes
Vaisman 2011 [42]	USA	84	45 ± 13	1986–2005	Retrospective cohort study	72	217	0.5–34 Y	Recurrence, DSS	no	PTC, HCC	no
Nixon 2012 [43]	USA	79	46	1986–2005	Retrospective cohort study	361	528	13 mo–24.3 Y	Recurrence, OS, DSS	yes	PTC, FTC, HCC	yes
Ebina 2014 [18]	Japan	76	54 ± 14	1993–2010	Retrospective cohort study	791	176	3–20 Y	Recurrence, DFS, DSS	yes	PTC	yes ***
Adam 2014 [44]	USA	80	NA	1998–2011	Retrospective population-based cohort	6849	54,926	5–14.9 Y	OS	yes	PTC	yes
Kuba 2017 [45]	Japan	86	53	1994–2008	Retrospective matched cohort study	120	53	11 mo–20.8 Y	OS, DFS	yes	PTC	yes

Percentage was rounded up to a closest integer. Y: years, mo: months, TT: total thyroidectomy, TL: thyroid lobectomy, OS: overall survival, DFS: disease free survival, DSS: disease specific survival, RAI: radioactive iodine, PTC: papillary thyroid carcinoma, FTC: follicular thyroid carcinoma, HCC: Hürthle cell carcinoma, NA: not available; * ”only a few received RAI” (not otherwise specified). ** The total cohort included 18% high-risk patients, however separate analysis available for the low-risk cohort, therefore only those patients were included in the meta-analysis. *** The total cohort included 19% high-risk patients, however separate analysis available for the low-risk cohort, therefore only those patients were included in the meta-analysis.

## Supplementary Materials

The following are available online at https://www.mdpi.com/2077-0383/9/7/2316/s1, Table S1: A comparison between scoring systems applied in included studies, Table S2: Comparison of the AJCC UICC 3rd, 6th, 7th and 8th edition, Table S3: Search strategy, Table S4: Radioactive iodine administration and inclusion of high-risk patients, Table S5: Risk of bias for each included study.
