# Supplementary material for: The Impact of the Extent of Surgery on the Long-Term Outcomes of Patients with Low-Risk Differentiated Non-Medullary Thyroid Cancer: A Systematic Meta-Analysis
Source: J Clin Med. 2020 Jul 21;9(7):2316. doi: 10.3390/jcm9072316 (PMC7408649; doi:10.3390/jcm9072316)
Supplement: Supplementary file 1 [file jcm-09-02316-s001.zip › Supplementary table 1,2.docx]

| **Table S1.** A comparison between different scoring systems applied in included studies^1^[20],^2^[22],^8^[23] | | | |
| --- | --- | --- | --- |
|  | **AJCC/UICC TNM** | **AMES** | **ATA** |
| **Histology** | All | DTC | PTC/FTC |
| **Grade** | no | no | no |
| **Age** (years) | <45 | <41 M  <51 F | no |
| **Gender** | no | yes | no |
| **Size** (cm) | ≤1  1-4  >4 | <5  ≥5 | no^a^ |
| **ETE** | yes | yes | yes |
| **Residual disease** | no | no | yes |
| **Lymph node** | yes | no | yes |
| **Multifocality** | no | no | yes |
| **Metastasis** | yes | yes | yes |
| ETE: extrathyroidal extension, DTC: differentiated thyroid cancer, PTC: papillary thyroid cancer, FTC: follicular thyroid cancer, M: male, F: female  ^a^No specific tumor size (intrathyroid tumor) | | | |

| **Table S2.** Comparison between 3^rd^, 6^th^, 7^th^ and 8^th^ Editions of AJCC/TNM staging of differentiated thyroid cancer^3^[23], [24], [25] | | | | | |
| --- | --- | --- | --- | --- | --- |
|  | **Stage** | **3^rd^ Edition** | **6^th^ Edition** | **7^th^ Edition** | **8^th^ Edition** |
| **Younger patients** |  | **<45 years** | | | **<55 years** |
|  | **I** | Any T, Any N, M0  Regardless of ETE | Any T, Any N, M0  Regardless of ETE | Any T, Any N, M0  Regardless of ETE | Any T, Any N, M0  Regardless of ETE |
|  | **II** | Any T, Any N, M1 | Any T, Any N, M1 | Any T, Any N, M1 | Any T, Any N, M1 |
| **Older patients** |  | **≥45 years** | | | **≥55 years** |
|  | **I** | T1 N0 M0  ≤1 cm tumor  Confined to the thyroid | T1 N0 M0  ≤ 2 cm tumor  Confined to the thyroid | T1 N0 M0  ≤ 2 cm tumor  Confined to the thyroid | T1-2 N0/Nx M0  ≤4 cm tumor  Confined to the thyroid |
|  | **II** | T2-3 N0 M0  1-4/>4 cm tumor  Confined to the thyroid | T2 N0 M0  2-4 cm tumor  Confined to the thyroid | T2 N0 M0  2-4 cm tumor  Confined to the thyroid | T1-2 N1a/N1b M0  ≤4 cm tumor  Confined to the thyroid    Or >4 cm tumor  T3a N0/Nx/N1a/N1b M0  Confined to the thyroid  Or Any T, Any N, M0  Gross ETE into strap muscles |
|  | **III** | T4 N0 M0  Any T, N1, M0  Any size beyond the thyroid capsule,  Or regional lymph node metastasis | T3 N0 M0  T1-3 N1a M0  >4 cm tumor,  Or minimal ETE,  Or level VI lymph  node metastasis | T3 N0 M0  T1-3 N1a M0  >4 cm tumor,  Or minimal ETE,  Or level VI lymph node metastasis | Any T, Any N, M0  Gross ETE into Subcutaneous, larynx, trachea, esophagus,  recurrent laryngeal  nerve |
|  | **IV** | Any T, Any N0, M1 | T4a N0-1 M0  T4b N0 M0  Any T N1b M0  Gross ETE or lateral neck lymph node metastasis,  Any T, Any N0, M1 | T4a N0-1 M0  Any T N1b M0  Gross ETE  Or lateral neck lymph node metastasis,  Any T, Any N0, M1 | **IVA:** Any T, Any N, M0  Gross ETE into prevertebral fascia, encasing major vessels    **IVB:** Any T, Any N, M1 |
| T: tumor, N: Node, M: metastasis, ETE: extrathyroidal extension | | | | | |

**References**

23. Haugen, B.R.; Alexander, E.K.; Bible, K.C.; Doherty, G.M.; Mandel, S.J.; Nikiforov, Y.E.; Pacini, F.; Randolph, G.W.; Sawka, A.M.; Schlumberger, M.; et al. 2015 American Thyroid Association Management Guidelines for adult patients with thyroid nodules and differentiated thyroid cancer: The American thyroid association guidelines task force on thyroid nodules and differentiated thyroid cancer. *Thyroid* **2016**, *26*, 1–133.

25. Cady, B.; Rossi, R. An expanded view of risk-group definition in differentiated thyroid carcinoma. *Surgery* **1988**, *104*, 947–953.

26. Tuttle, R.M.; Haugen, B.; Perrier, N.D. Updated American Joint Committee on Cancer/Tumor-Node-Metastasis Staging System for Differentiated and Anaplastic Thyroid Cancer (Eighth Edition): What Changed and Why? *Thyroid* **2017**, *27*, 751–756, doi:10.1089/thy.2017.0102.

27. Bearhs, O.H.; Henson, D.E. *AJCC Manual for Staging Cancer*, 3rd ed.; J.B. Lippincott Company: Philadelphia, PA, USA, 1988; pp. 61–62.

28. Greene, F.I.; Page, D.L. *AJCC Manual for Staging Cancer*, 6th ed.; Springer: New York, NY, USA, 2002; pp. 77–79.
