# Supplementary material for: The Impact of the Extent of Surgery on the Long-Term Outcomes of Patients with Low-Risk Differentiated Non-Medullary Thyroid Cancer: A Systematic Meta-Analysis
Source: J Clin Med. 2020 Jul 21;9(7):2316. doi: 10.3390/jcm9072316 (PMC7408649; doi:10.3390/jcm9072316)
Supplement: Supplementary file 1 [file jcm-09-02316-s001.zip › Supplementary table 3. Search strategy.docx]

**Table S3.** Search strategy

(total[All Fields] AND ("thyroidectomy"[MeSH Terms] OR "thyroidectomy"[All Fields])) AND (lobectomy[All Fields] NOT ("lung"[MeSH Terms] OR "lung"[All Fields]))) AND (("thyroid gland"[MeSH Terms] OR ("thyroid"[All Fields] AND "gland"[All Fields]) OR "thyroid gland"[All Fields] OR "thyroid"[All Fields] OR "thyroid (usp)"[MeSH Terms] OR ("thyroid"[All Fields] AND "(usp)"[All Fields]) OR "thyroid (usp)"[All Fields]) AND ("surgery"[Subheading] OR "surgery"[All Fields] OR "surgical procedures, operative"[MeSH Terms] OR ("surgical"[All Fields] AND "procedures"[All Fields] AND "operative"[All Fields]) OR "operative surgical procedures"[All Fields] OR "surgery"[All Fields] OR "general surgery"[MeSH Terms] OR ("general"[All Fields] AND "surgery"[All Fields]) OR "general surgery"[All Fields]))) AND ((low[All Fields] AND ("risk"[MeSH Terms] OR "risk"[All Fields]) AND ("thyroid neoplasms"[MeSH Terms] OR ("thyroid"[All Fields] AND "neoplasms"[All Fields]) OR "thyroid neoplasms"[All Fields] OR ("thyroid"[All Fields] AND "carcinoma"[All Fields]) OR "thyroid carcinoma"[All Fields])) OR (("thyroid gland"[MeSH Terms] OR ("thyroid"[All Fields] AND "gland"[All Fields]) OR "thyroid gland"[All Fields] OR "thyroid"[All Fields] OR "thyroid (usp)"[MeSH Terms] OR ("thyroid"[All Fields] AND "(usp)"[All Fields]) OR "thyroid (usp)"[All Fields]) AND non-medullary[All Fields] AND ("neoplasms"[MeSH Terms] OR "neoplasms"[All Fields] OR "cancer"[All Fields])
