# Supplementary material for: The Impact of the Extent of Surgery on the Long-Term Outcomes of Patients with Low-Risk Differentiated Non-Medullary Thyroid Cancer: A Systematic Meta-Analysis
Source: J Clin Med. 2020 Jul 21;9(7):2316. doi: 10.3390/jcm9072316 (PMC7408649; doi:10.3390/jcm9072316)
Supplement: Supplementary file 1 [file jcm-09-02316-s001.zip › Supplementary table 4. RAI and HR.docx]

| **Table S4.** Radioactive iodine administration and inclusion of high-risk patients. | | | | | | | | | | |
| --- | --- | --- | --- | --- | --- | --- | --- | --- | --- | --- |
| **Study** | **RAI given** | **HR included**  **(% of total)** | **Risk stratification system** | **Total thyroidectomy** | | | **Thyroid lobectomy** | | | **Separate analysis for RAI** |
|  |  |  |  | **N** | **Of which HR (%)** | **RAI (%^*****^)** | **N** | **Of which HR** | **RAI (%)** |  |
| Samaan 1983 | yes (34%) | yes (4.6% M+) | None | 491 | NA | NA | 105 | NA | NA | yes |
| Shah 1993 | yes^*^ | yes | AJCC 3^rd^ ed. | 73 | NA (8% M+) | NA | 73 | NA (8% M+) | NA | no |
| Shaha 1997 | NA | no | AJCC 3^rd^ ed. | 90 | 0% | NA | 375 | 0% | NA | no |
| Hay 1998 | yes | no | AMES | 1451 | 0% | 32% | 187 | 0% | 3.0% | yes |
| Sanders 1998 | NA | yes (22.0%) | AMES | 795 | NA | NA | 224 | NA | NA | yes |
| Haigh 2004 | yes | yes (19%)^**^ | AMES | 3663 | 0% | 48% | 739 | 0% | 12% | yes |
| Bilimoria 2007 | yes | Yes | None | 43227 | NA (2.4% M+) | 56.2% | 8946 | NA (1.2% M+) | 18.4% | no |
| Mendelsohn 2010 | yes | Yes | None | 16760 | NA (1.2% M+) | 54.4% | 5964 | NA (0.5% M+) | 19.9% | yes |
| Hassanain 2010 | yes (28.3%) | yes (15%) | AJCC 6^th^ ed./  AMES/MACIS | 54 | 48.1% | NA (94.4%) | 126 | 0% | 0% | no |
| Ito 2010 | yes (0.1%) | no | AJCC 7^th^ ed. | 1037 | 0% | NA | 1601 | 0% | NA | no |
| Barney 2011 | yes | yes | AJCC 6^th^ ed. | 12598 | NA (1.8% M+) | 54.5% | 3266 | NA (0.6% M+) | 11.8% | no |
| Vaisman 2011 | no | no | AJCC 6^th^ ed./  ATA 2009 | 219 | 0% | 0% | 72 | 0% | 0% | no RAI |
| Nixon 2012 | yes | yes (7%) | AJCC 6^th^ ed. | 528 | NA | 37% | 361 | NA | 0.3% | no |
| Ebina 2014 | yes | yes (18%)^***^ | AJCC 7^th^ ed. | 238 | NA | NA | 728 | NA | NA | no |
| Adam 2014 | yes | yes | AJCC 7^th^ ed. | 54926 | NA (0.4% M+) | 65% | 6849 | NA (1.1% M+) | 33% | yes |
| Kuba 2017 | yes | yes (49%) | AJCC 7^th^ ed. | 53 | 46%^****^ | 7% | 120 | 39%^****^ | 0% | no |
| RAI: radioactive iodine, HR: high-risk, N: number, NA: not available, M+: distant metastases  ^*^ ”only a few received RAI” (not otherwise specified)  ^**^ The total cohort included 19% high-risk patients, however separate analysis available for the low-risk cohort, therefore only those patients were included in the meta-analysis.  ^***^ The total cohort included 18% high-risk patients, however separate analysis available for the low-risk cohort, therefore only those patients were included in the meta-analysis.  ^****^ Stage III and IV (AJCC 7^th^ edition)  ^*****^ Including for high-risk patients if they were included in the series. | | | | | | | | | | |
